# *Stachys sieboldii Miq*. Root Attenuates Weight Gain and Dyslipidemia in Rats on a High-Fat and High-Cholesterol Diet

**DOI:** 10.3390/nu12072063

**Published:** 2020-07-11

**Authors:** Jennifer K. Lee, Jae-Joon Lee, Yeon-Kyoung Kim, Youngseung Lee, Jung-Heun Ha

**Affiliations:** 1 Food Science & Human Nutrition Department, University of Florida, Gainesville, FL 32611, USA; leejennifer@ufl.edu; 2 Department of Food and Nutrition, Chosun University, Gwangju 61452, Korea; leejj80@chosun.ac.kr (J.-J.L.); kyg-juli4367@hanmail.net (Y.-K.K.); 3 Research Center for Industrialization of Natural Neutralization, Dankook University, Cheonan 31116, Korea; 4 Department of Food Science and Nutrition, Dankook University, Cheonan 31116, Korea

**Keywords:** *Stachys sieboldii Miq*, anti-obesity, cholesterol-lowering, high-fat and high-cholesterol (HFC) diet

## Abstract

This study aimed at investigating the anti-obesity and anti-dyslipidemic effects of *Stachys sieboldii Miq*. root (SS) powder in rats following a high-fat and high-cholesterol (HFC) diet for 6 weeks. Thirty-two Sprague–Dawley rats were fed one of the following diets: a regular diet (RD), HFC, HFC supplemented with 3% SS (HFC + 3SS) or HFC supplemented with 5% SS (HFC + 5SS). Following an HFC diet increased body weight (BW) gain (*p* < 0.001) and the food efficiency ratio (FER; *p* < 0.001); however, SS consumption gradually prevented the HFC-induced BW gain (*p* < 0.001) and increase in FER (*p* < 0.01). The HFC diet resulted in increased liver size (*p* < 0.001) and total adipose tissue weight (*p* < 0.001), whereas the SS supplementation decreased hepatomegaly (*p* < 0.05) and body fat mass (*p* < 0.001). SS consumption prevented the increased activities of serum alanine aminotransferase (ALT; *p* < 0.001), aspartate aminotransferase (AST; *p* < 0.001), alkaline phosphatase (ALP; *p* < 0.01 in HFC + 5SS) and lactate dehydrogenase (LDH; *p* < 0.001 in HFC + 5SS) induced by the HFC diet (*p* < 0.001). The SS supplementation improved lipid profiles in the circulation by lowering triglyceride (TG; *p* < 0.01), total cholesterol (TC; *p* < 0.001) and non-HDL cholesterol (non-HDL-C; *p* < 0.001) levels, as well as the atherogenic index (*p* < 0.01) and cardiac risk factor (*p* < 0.01). The lipid distribution in the liver (*p* < 0.05) and white adipose tissues (WAT; *p* < 0.001) of the HFC + SS diet-consuming rats was remarkably lower than that of the HFC diet-consuming rats. The average size of the epididymal adipose tissue (*p* < 0.001) was significantly lower in the HFC + SS diet-fed rats than in the HFC diet-fed rats. The fecal lipid (>3% SS; *p* < 0.001) and cholesterol (5% SS; *p* < 0.001) efflux levels were significantly elevated by the SS supplementation compared to those measured in the RD or HFC diet-fed groups. In addition, the hepatic lipid and cholesterol metabolism-related gene expressions were affected by SS consumption, as the hepatic anabolic gene expression (Acc; *p* < 0.001, Fas; *p* < 0.001 and G6pdh; *p* < 0.01) was significantly attenuated. The HFC + 5SS diet-fed rats exhibited elevated hepatic Cyp7a1 (*p* < 0.001), Hmgcr (*p* < 0.001) and Ldlr (*p* < 0.001) mRNA expression levels compared to the HFC diet-fed rats. These results suggest that SS may possess anti-adipogenic and lipid-lowering effects by enhancing lipid and cholesterol efflux in mammals.

## 1. Introduction

Obesity has become a major health problem worldwide. Over the past decades, the prevalence of obesity has increased dramatically. In 2017–2018 in the United States, approximately 42.4% of adults were obese [1]. Obesity is a major contributor to the development of health complications, including insulin resistance, hypertension, alterations in lipid metabolism and metabolic syndrome (MetS) [2,3]. In particular, excess abdominal fat is strongly related to metabolic diseases [4]. Excessive fat accumulation in obesity leads to dyslipidemia, which is a major risk factor for cardiovascular disease (CVD) [5]. Obesity-related dyslipidemia is associated with increased triglyceride, apolipoprotein B and non-HDL cholesterol levels [6]. Thus, the prevention and treatment of obesity-associated dyslipidemia through weight reduction may have beneficial effects on the overall risk factors for metabolic diseases.

Lifestyle modification (such as a well-balanced diet and exercise) is the most important therapeutic strategy to improve the health conditions of patients with insulin resistance, obesity and dyslipidemia [7]. However, lifestyle intervention alone may not be sufficient to induce clinically meaningful weight loss [8]. Given the limitations of lifestyle modifications, pharmacological approaches for the treatment of atherogenic dyslipidemia offer a possible option [9]. Statins are the widest-prescribed drugs to reduce LDL-C, non-HDL-C, and/or apo B levels [10]. While statins are first-line drugs, they do not adequately correct atherogenic dyslipidemia, residual cardiovascular risk thus remains high [11]. Statins competitively inhibit 3-hydroxy-3-methylglutaryl-coenzyme A (HMG-CoA) reductase, which is the rate-limiting enzyme in the cholesterol biosynthesis pathway [12]. Despite the widespread use of statins, their long-term use has become a concern due to their side effects [13]. Statin-associated muscle symptoms (SAMS) are the most common side effects of statins, including myalgia, cramps and weakness [14]. While the mechanisms of SAMS are not completely understood, one possible hypothesis states that statins may decrease coenzyme Q10 (CoQ10) synthesis [13]. The depletion of CoQ10 within muscles could alter mitochondrial function and cellular energy use, resulting in decreased ATP production and subsequent cell damage [15]. Other side effects include new-onset type 2 diabetes mellitus, neurological and neurocognitive effects, hepatic dysfunction, renal insufficiency and serious infections [12,16]. Since several synthetic drugs for the treatment of obesity-related dyslipidemia have reported toxic- and side effects, the use of plant-based natural products, as safer alternatives, are of great interest.

*Stachys* L. (Lamiaceae) is one of the largest genera of Labiate, containing an estimated 300 species [17]. *Stachys sieboldii* is a widely used folk medicine in China, as well as food ingredients in Korea and Japan due to its therapeutic benefits, including anti-inflammatory, antitoxic and antibacterial activities [18,19]. To date, there have been no reported harmful effects associated with *Stachys sieboldii*. Being a great source of oligosaccharides, proteins and water-soluble vitamins, *S. sieboldii* Miq. root (SS) has been used to treat the common cold, heart disease, urinary tract infections, tuberculosis, etc. [18,20]. However, the therapeutic potential and the underlying effector mechanisms of SS in obesity-related dyslipidemia remain largely unknown. In this study, we investigated the anti-obesity and anti-dyslipidemic effects of the SS powder in rats, following a high-fat and high-cholesterol diet (HFC). Furthermore, we investigated by blood, liver and adipose tissue sample collection how SS affects lipid metabolism.

## 2. Materials and Methods

### 2.1. Material Preparation

The SS root powder used in this experiment was produced in Hadong-gun, Gyeongsangnam-do in July 2016, and was purchased from Jirisan Chacheonji. The roots were washed three times with tap water in order to remove sand and dust attached to the surface. The rinsed SS roots were freeze-dried for 72 h and powdered by grinding. The SS powder was then stored at −70 °C until the experimental rodent diets were fabricated. Total polyphenol content (~20.44 mg TAE/g), total flavonoid content (~11.51 mg RE/g), and other nutritional components of the SS root powder were previously described [21].

### 2.2. Animal Experiments and Diets

All animal studies were approved by the Chosun University Institutional Animal Care and Use Committee (C IACUC 2016-A0019). After 1 week of acclimatization, 5-week old male Sprague-Dawley rats (Orient Bio, Inc.; Seongnam-Si, Korea) were housed in shoebox cages for 6 weeks until sacrifice. The experimental animals were housed at a temperature of 18 ± 2 °C, humidity of 55% ± 5%, and with a 12-h light–dark cycle (8 AM–8 PM) at the Center for Animal Experiment, Chosun University. The animals had free access to food and water during the experimental period. The experimental animals were randomly assigned to one of the four experimental groups (*n* = 8 per group). The experimental rats in each group were fed either a regular diet (RD), based on the AIN-93G formulation, (15.8% of fat-derived calories), a high-fat and high-cholesterol diet (HFC, 35.6% of fat-derived calories), a HFC diet supplemented with 3% of SS (HFC + 3SS, 36.6% of fat-derived calories) or a HFC diet supplemented with 5% of SS (HFC + 5SS, 37.2% of fat-derived calories) for 6 weeks. The detailed composition of the experimental diet is shown in Table 1. The body weight (BW) and food intake were measured weekly. The food efficiency ratio (FER) was calculated as follows: total BW gain/total food intake. At the end of the study, whole blood samples were collected from the abdominal vein and isolated as serum. The liver and adipose tissues were harvested and weighed after sacrifice by thoracotomy after CO_2_ narcosis. The harvested organs and serum samples were stored at −80 °C until further analysis.

### 2.3. Biochemical Analysis of Serum Samples

The enzymatic activities of serum aspartate aminotransferase (AST), alanine aminotransferase (ALT), alkaline phosphatase (ALP), lactate dehydrogenase (LDH), triglyceride (TG), total cholesterol (TC), high-density lipoprotein cholesterol (HDL-C) and fasting glucose (Glu) levels were measured as previously described [22] using a Chemistry Analyzer (Fujifilm Dri-Chem 3500i, Fujifilm, Tokyo, Japan). The concentration of non-high-density lipoprotein cholesterol (non-HDL-C) was calculated by subtracting HDL-C from TC. Atherogenic index (AI = (TC − HDL-C)/HDL-C) and cardiac risk factor (CRF = TC/HDL-C) were calculated [23].

### 2.4. Tissue and Fecal Lipid Contents

Lipids were extracted from ~0.1 g of liver and adipose tissues as previously described [24]. The feces were collected 3 days before sacrifice and lyophilized in a deep freezer. The total fecal lipid was extracted from 500-mg of fecal samples using a well-established method [24]. The TG and TC were measured from the lower (lipids-abundant) layer following previous methods [25,26].

### 2.5. Reverse Transcription Polymerase Chain Reaction (RT-PCR)

The total RNA samples were extracted using the RNeasy mini kit (QIAGEN, Germantown, MD, USA) according to the manufacturer’s protocol. The RT-PCR was performed as previously described [27]. The expression of each experimental gene was normalized to the expression of β-actin, which did not significantly vary between the different dietary settings. Mean fold changes in mRNA expression were calculated by the 2^−ΔΔCt^ analytical method. The gene-specific oligonucleotide primers that were used in this study are available in Table 2.

### 2.6. Histological Analysis

Liver and epididymal adipose tissues were fixed, sectioned and stained as described previously [22].

### 2.7. Statistical Analysis

The experimental data were analyzed using a one-way analysis of variance (ANOVA; GraphPad PRISM 8, San Diego, CA, USA). Subsequently, Tukey’s post hoc test was applied to distinguish groups that varied significantly at least *p* < 0.05.

## 3. Results

### 3.1. SS Consumption Ameliorated HFC-Induced Weight Gain

This study was conducted to evaluate the obesity- and dyslipidemia-preventive effects of SS using HFC-inducible obese and dyslipidemic rat models. Male Sprague-Dawley rats in each group were fed either an RD, an HFC, an HFC + 3SS or an HFC + 5SS diet for 6 weeks. The dietary compositions are summarized in Table 1.

**Figure 1:**
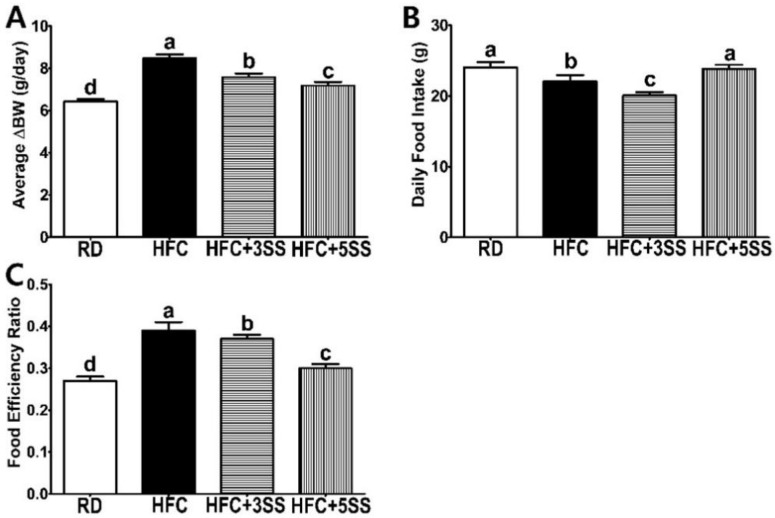
Effects of *Stachys sieboldii Miq.* root powder (SS) on body weight gain, food intake and food efficiency ratio in HFC diet-fed rats. Experimental rats were fed a regular diet (RD) or a high-fat and high-cholesterol (HFC) diet with or without *Stachys sieboldii Miq.* root powder (SS; 3 or 5%) supplementation for 6 weeks. (**A**) Delta body weight gain (the final BW after dietary feeding—the initial BW), (**B**) daily food consumption and (**C**) food efficiency ratio were measured. RD, regular diet; HFC, high-fat and high-cholesterol diet; HFC + 3SS, High-fat and high-cholesterol diet + 3% of *Stachys sieboldii Miq. root* powder; HFC + 5SS, high-fat and high-cholesterol diet + 5% of *Stachys sieboldii Miq.* root powder. Values are means ± standard deviation, *n* = 8. Data were analyzed using one-way ANOVA followed by Tukey’s post hoc test. Means labeled without a common letter differ significantly, *p* < 0.05.

The results showed that both the BW gain and the final BW (after dietary feeding minus the initial BW) were significantly higher in the HFC group than in the RD group. However, the SS supplementation gradually prevented weight gain in a dose-dependent manner (Figure 1A). The HFC diet-fed rats consumed less food than the RD-fed animals, whereas the HFC + 3SS diet-fed rats had the lowest daily food intake (Figure 1B). Interestingly, HFC + 5SS diet-fed rats consumed a higher daily amount of food than the animals in the HFC or HFC + 3SS groups (Figure 1B), while the BW gain of this group was markedly lower than those of the HFC or HFC + 3SS diet-fed animals (Figure 1A). According to the FER assessment, the RD feeding resulted in the lowest, while the HFC diet feeding resulted in the highest FER levels (Figure 1C). However, increasing the SS consumption significantly decreased FER levels in a dose-dependent manner (Figure 1C). Therefore, it could be postulated that SS consumption inhibited the HFC diet-induced FER level increase.

**Figure 2:**
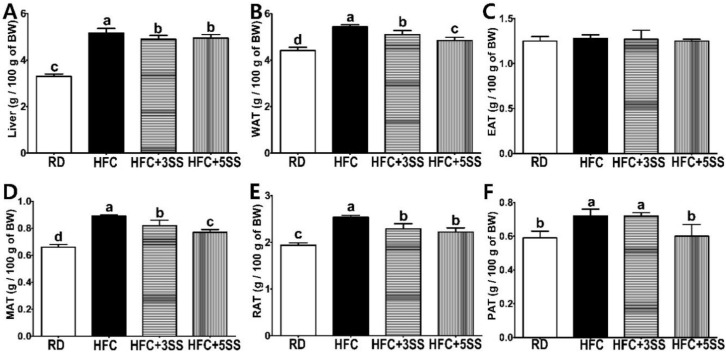
Effects of *Stachys sieboldii* Miq. root powder (SS) on hepatomegaly and adiposity. Experimental rats were fed a regular diet (RD) or a high-fat and high-cholesterol (HFC) diet with or without *Stachys sieboldii Miq*. root powder (SS; 3 or 5%) supplementation for 6 weeks. (**A**) Relative liver, (**B**) white adipose tissue (WAT), (**C**) epididymal adipose tissue (EAT), (**D**) mesenteric adipose tissue (MAT), (**E**) retroperitoneal adipose tissue (RAT) and (**F**) perirenal adipose tissue (PAT). Relative tissue weights were calculated as g/100 g of BW. RD, regular diet; HFC, high-fat and high-cholesterol diet; HFC + 3SS, High-fat and high-cholesterol diet + 3% of *Stachys sieboldii Miq*. root powder; HFC + 5SS, high-fat and high-cholesterol diet + 5% of *Stachys sieboldii Miq*. root powder. Values are means ± standard deviation, *n* = 8. Data were analyzed using one-way ANOVA followed by Tukey’s post hoc test. Means labeled without a common letter differ significantly, *p* < 0.05.

To examine the weight change in the representative metabolic organs by feeding male rats an HFC diet, the liver and multiple white adipose tissues (WAT) were weighed and compared. We observed hepatomegaly in all HFC diet-fed rats. However, the SS-supplemented HFC diet attenuated hepatomegaly compared to the liver weight measured in the HFC group (Figure 2A). As expected, the HFC group had the highest total WAT mass, whereas the HFC + 3SS and HFC + 5SS groups exhibited a dose-dependently decreasing total WAT mass (Figure 2B). Among the WAT tissues, the weight of the epididymal adipose tissue (EAT) did not vary among the different dietary conditions (Figure 2C). Interestingly, the relative weight of the mesenteric adipose tissues (MAT) reflected the most statistically dynamic alteration (HFC > HFC + 3SS > HFC + 5SS > RD, showed in Figure 2D). The relative weight of the retroperitoneal adipose tissues (RAT) decreased with SS consumption compared to that measured in the HFC group (HFC > HFC + 3SS = HFC + 5SS > RD; Figure 2E). The relative weight of the perirenal adipose tissue (PAT) was increased by the HFC diet (Figure 2F). The weight of the PAT in the HFD + 3SS group remained similar to the weight measured in the HFC group. However, the weight of the PAT was lower in the RD and HFC + 5SS groups than in the HFC or HFC + 3SS groups.

### 3.2. Hepatic Function Tests and Fasting Glucose Levels

**Figure 3:**
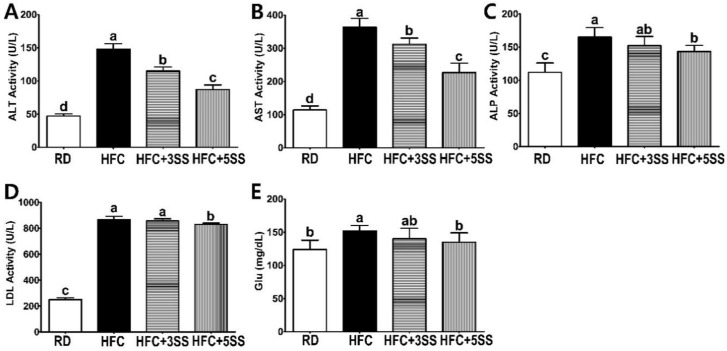
Effect of *S. sieboldii* Miq. root powder (SS) on hepatic function. Experimental rats were fed a regular diet (RD) or a high-fat and high-cholesterol (HFC) diet with or with-out *Stachys sieboldii* Miq. root powder (SS; 3 or 5%) supplementation for 6 weeks. (**A**) Alanine aminotransferase (ALT), (**B**) aspartate aminotransferase (AST), (**C**) alkaline phosphatase (ALP), (**D**) lactate dehydrogenase (LDH) and (**E**) fasting glucose (Glu) were analyzed enzymatically or biochemically. RD, regular diet; HFC, high-fat and high-cholesterol diet; HFC + 3SS, high-fat and high-cholesterol diet + 3% of *Stachys sieboldii* Miq. root powder; HFC + 5SS, high-fat and high-cholesterol diet + 5% of *Stachys sieboldii* Miq. root powder. Values are means ± standard deviation, *n* = 8. Data were analyzed by one-way ANOVA followed by Tukey’s post hoc test. Means labeled without a common letter differ significantly, *p* < 0.05.

To evaluate the effect of SS on the hepatic function and fasting glucose levels 6 weeks after HFC diet feeding, the AST, ALT, ALP and LDH enzymatic activities, as well as the fasting glucose levels were examined in the serum (Figure 3). All results in the HFC group were significantly higher compared to the RD group. The elevated HFC diet-induced ALT (Figure 3A) and AST (Figure 3B) activities were significantly prevented by SS supplementation in a dose-dependent manner (HFC > HFC + 3SS > HFC + 5SS > RD). The ALP (Figure 3C) and LDH (Figure 3D) activity inductions were partially prevented in the HFC + 5SS group compared to the HFC group. The fasting glucose (Glu, showed in Figure 3E) levels were significantly lower in the HFC + 5SS (135 mg/dL) group compared to those in the HFC (152 mg/dL) group. Notably, the Glu level in the HFC + 5SS group was similar to that observed in the RD group (124 mg/dL, showed in Figure 3E). In summary, SS supplementation significantly prevented hepatic function impairment and HFC-induced fasting Glu level increase.

### 3.3. Serum Lipid Profiles

**Figure 4:**
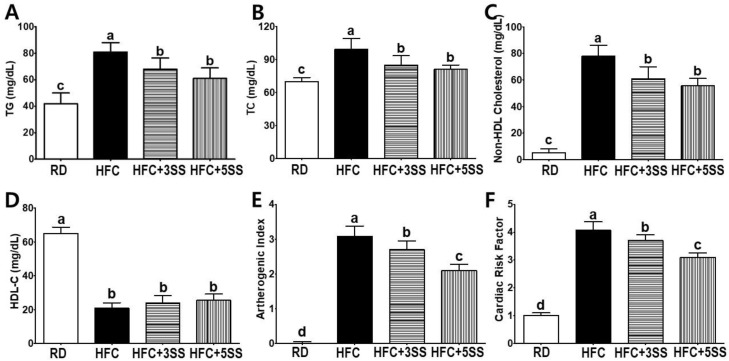
Effect of *S. sieboldii* Miq. root powder (SS) on lipid profiles and cardiovascular indices. Experimental rats were fed a regular diet (RD) or a high-fat and high-cholesterol (HFC) diet with or without *Stachys sieboldii* Miq. root powder (SS; 3 or 5%) supplementation for 6 weeks. Serum levels of (**A**) triglyceride (TG), (**B**) total cholesterol (TC), (**C**) non-high-density lipoprotein cholesterol (non-HDL-C), (**D**) high-density lipoprotein cholesterol (HDL-C), (**E**) atherogenic index (AI) and (**F**) cardiac risk factor (CRF) were measured in the experimental rats. RD, regular diet; HFC, high-fat and high-cholesterol diet; HFC + 3SS, High-fat and high-cholesterol diet + 3% of *Stachys sieboldii* Miq. root powder; HFC + 5SS, high-fat and high-cholesterol diet + 5% of *Stachys sieboldii* Miq. root powder. Values are means ± standard deviation, *n* = 8. Data were analyzed using one-way ANOVA followed by Tukey’s post hoc test. Means labeled without a common letter differ significantly, *p* < 0.05.

In order to elucidate the preventive effects of SS in the HFC diet-induced imbalanced lipid metabolism, we carefully assessed the CVD-related lipid profile alterations in the serum. The HFC group demonstrated significantly higher levels of TG (Figure 4A), TC (Figure 4B) and non-HDL-C (Figure 4C) compared to the RD group. As per our expectation, TG, TC and non-HDL-C levels were attenuated following SS consumption compared to the HFC group (Figure 4A–C). The HDL-C levels were higher in the RD group than in the other groups (Figure 4D). Based on the previously analyzed lipid profiles, we processed the AI and CRF values. Both the AI (Figure 4E) and CRF (Figure 4F) levels were higher in the HFC group than in the RD group. Both CVD-related indices were gradually attenuated with SS supplementation (HFD + 3SS and HFD + 5SS) in a dose-dependent manner (Figure 4E,F). Our findings demonstrated that oral SS consumption could significantly recuperate lipid profiles and possible CVD-onset risk in HFC diet-fed rats.

### 3.4. Liver and WAT Lipid Levels

**Figure 5:**
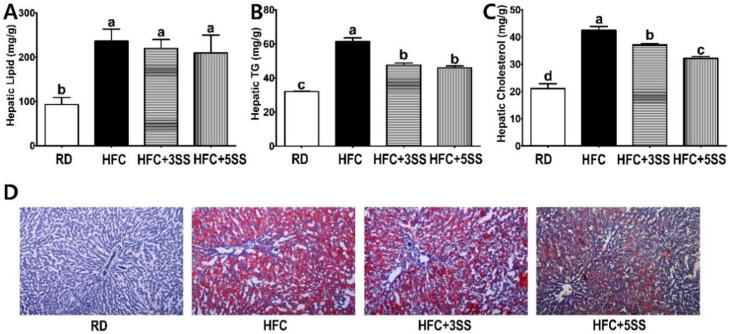
Effect of *S. sieboldii* Miq. root powder (SS) on hepatic lipid accumulation. Experimental rats were fed a regular diet (RD) or a high-fat and high-cholesterol (HFC) diet with or without *Stachys sieboldii* Miq. root powder (SS; 3 or 5%) supplementation for 6 weeks. (**A**) Hepatic lipid, (**B**) hepatic triglyceride (TG) and (**C**) hepatic cholesterol were measured and expressed as mg/g of tissue weight; (**D**) liver samples from each experimental group were harvested, fixed and stained with Oil red O. RD, regular diet; HFC, high-fat and high-cholesterol diet; HFC + 3SS, high-fat and high-cholesterol diet + 3% of *Stachys sieboldii* Miq. root powder; HFC + 5SS, high-fat and high-cholesterol diet + 5% of *Stachys sieboldii* Miq. root powder. Values are means ± standard deviation, *n* = 8. Data were analyzed using one-way ANOVA followed by Tukey’s post hoc test. Means labeled without a common letter differ significantly, *p* < 0.05.

Based on the decreased circulatory lipid levels due to the SS supplementation, we logically postulated that such supplementation may also attenuate lipid deposits in the liver and WAT. Therefore, we measured the total lipid, TG and TC levels in the liver and WAT of HFC diet-fed experimental rats. In the liver, the total lipid accumulation was elevated with the HFC diet regardless of the SS supplementation (Figure 5A). However, SS supplementation significantly attenuated hepatic TG (Figure 5B) and TC (Figure 5C) accumulation compared to the HFC group. Interestingly, SS supplementation lowered the hepatic TC content in a dose-dependent manner (Figure 5C). In order to histologically determine the degree of hepatic neutral lipid accumulation, we performed an Oil Red O staining. Notably, the SS-supplied groups (HFC + 3SS and HFC + 5SS) deposited fewer neutral lipids in the liver tissues than the HFC group (Figure 5D).

**Figure 6:**
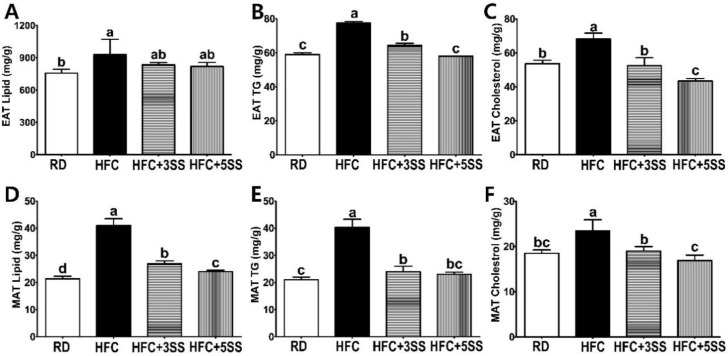
Effect of *S. sieboldii* Miq. root powder (SS) on adipocyte lipid accumulation. Experimental rats were fed a regular diet (RD) or a high-fat and high-cholesterol (HFC) diet with or without *Stachys sieboldii* Miq. root powder (SS; 3 or 5%) supplementation for 6 weeks. (**A**,**D**) Lipid, (**B**,**E**) triglyceride and (**C**,**F**) cholesterol were measured from the epididymal adipose tissue (EAT) and mesenteric adipose tissue (MAT) and expressed as mg/g. RD, regular diet; HFC, high-fat and high-cholesterol diet; HFC + 3SS, High-fat and high-cholesterol diet + 3% of *Stachys sieboldii* Miq. root powder; HFC + 5SS, high-fat/high-cholesterol diet + 5% of *Stachys sieboldii* Miq. root powder. Values are means ± standard deviation, *n* = 8. Data were analyzed using one-way ANOVA followed by Tukey’s post hoc test. Means labeled without a common letter differ significantly, *p* < 0.05.

**Figure 7:**
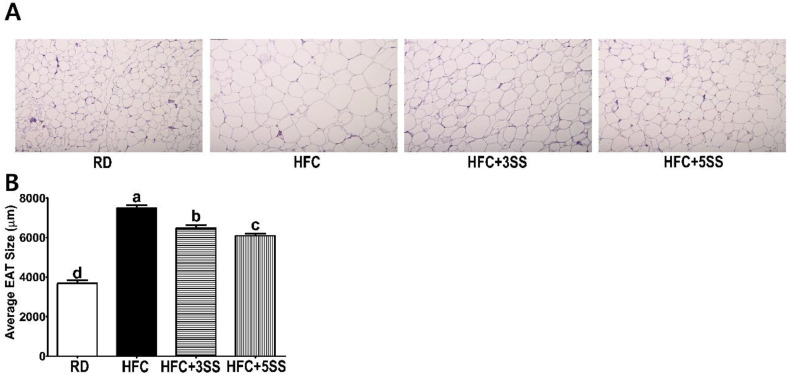
Effect of *S. sieboldii* Miq. root powder (SS) on adipocyte size. Experimental rats were fed a regular diet (RD) or a high-fat and high-cholesterol (HFC) diet with or without *Stachys sieboldii* Miq. root powder (SS; 3 or 5%) supplementation for 6 weeks. The epididymal adipose tissue (EAT) was stained with Hematoxylin and Eosin (HE). Magnification, 100×. The EAT surface area was quantified using the Image J program. (**A**) Representative EAT images; (**B**) Quantification of the surface area of EAT. RD, regular diet; HFC, high-fat and high-cholesterol diet; HFC + 3SS, High-fat and high-cholesterol diet + 3% of *Stachys sieboldii* Miq. root powder; HFC + 5SS, high-fat and high-cholesterol diet + 5% of *Stachys sieboldii* Miq. root powder. Values are means ± standard deviation, *n* = 8. Data were analyzed using one-way ANOVA followed by Tukey’s post hoc test. Means labeled without a common letter differ significantly, *p* < 0.05.

Among the WATs, we assessed the lipid levels in EAT and MAT. The rationale for selecting particular WATs was that the weight of EAT was not altered by SS supplementation (Figure 2C), whereas the weight of MAT was significantly reduced by it in a dose-dependent manner (Figure 2D). In the EAT, the total lipid levels exhibited a decreasing trend upon SS supplementation regardless of the concentration (Figure 6A). Intriguingly, the TG (Figure 6B) and TC (Figure 6C) levels in the EAT were significantly attenuated by the SS supplementation in a dose-dependent manner. The total lipid (Figure 6D), TG (Figure 6E) and TC (Figure 6F) contents in the MAT were increased in the HFC group. However, these lipid accumulations were significantly reduced by SS supplementation in a dose-dependent manner (Figure 6D–F). In addition, the SS supplementation (in the HFC + 3SS and HFC + 5SS groups) significantly reduced the EAT size (Figure 7A,B), although the total EAT weight did not significantly differ (Figure 2C).

### 3.5. Fecal Lipid Composition

**Figure 8:**
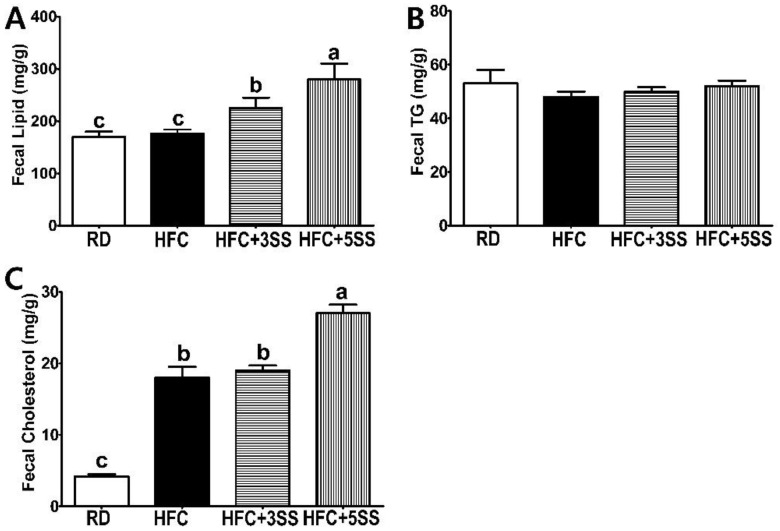
Effect of *S. sieboldii* Miq. root powder (SS) in fecal lipid excretion. Experimental rats were fed a regular diet (RD) or a high-fat and high-cholesterol (HFC) diet with or with-out *Stachys sieboldii* Miq. root powder (SS; 3 or 5%) supplementation for 6 weeks. (**A**) Fecal lipid, (**B**) fecal triglyceride (TG) and (**C**) fecal cholesterol were measured and expressed as mg/g. RD, regular diet; HFC, high-fat and high-cholesterol diet; HFC + 3SS, High-fat and high-cholesterol diet + 3% of *Stachys sieboldii* Miq. root powder; HFC + 5SS, high-fat and high-cholesterol diet + 5% of *Stachys sieboldii* Miq. root powder. Values are means ± standard deviation, *n* = 8. Data were analyzed using one-way ANOVA followed by Tukey’s post hoc test. Means labeled without a common letter differ significantly, *p* < 0.05.

The SS supplementation remarkably decreased the lipid levels in the blood, liver and adipose tissues of the HFC diet-fed male SD rats. Therefore, we hypothesized that the SS supplementation may enhance the fecal lipid excretion. In order to support our extended research hypothesis, we analyzed the levels of fecal lipids, TG and TC. The total lipid content in the fecal samples was not altered by HFC compared to the RD group (Figure 8A). However, as per our assumption, the SS supplementation dramatically increased the fecal lipid excretion in a dose-dependent manner (Figure 8A). The fecal TG content was not affected by any of the different dietary assignments (Figure 8B). The HFC-induced fecal cholesterol excretion was escalated compared to that in the RD group due to the HFC-contained higher dietary cholesterol (Figure 8C). The SS supplementation may intensify the fecal cholesterol excretion as this value was higher in the HFC + 5SS diet-fed rats compared to the HFC diet-fed rats.

### 3.6. Hepatic Lipid Metabolism-Related mRNA Expression

**Figure 9:**
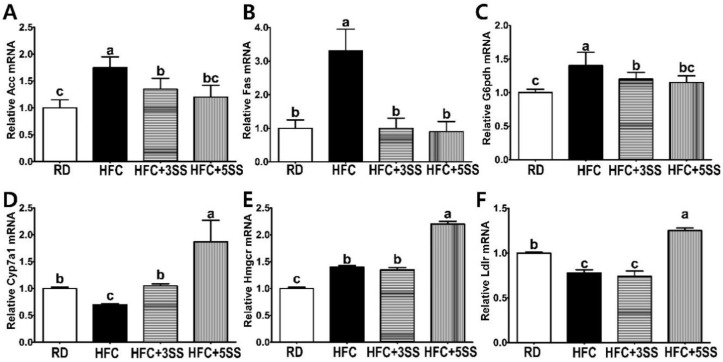
Effect of *S. sieboldii* Miq. root powder (SS) on hepatic mRNA expression. Experimental rats were fed a regular diet (RD) or a high-fat and high-cholesterol (HFC) diet with or without *Stachys sieboldii* Miq. root powder (SS; 3 or 5%) supplementation for 6 weeks. Hepatic (**A**) acetyl-CoA carboxylase (ACC), (**B**) fatty acid synthase (FAS), (**C**) Glucose-6-phosphate dehydrogenase (G6pdh), (**D**) cholesterol 7 alpha-hydroxylase (Cyp7a1), (**E**) 3-hydroxy-3-methyl-glutaryl-coenzyme A reductase (Hmgcr) and (**F**) low-density lipoprotein (LDL) receptor (Ldlr) mRNA expression. RD, regular diet; HFC, high-fat and high-cholesterol diet; HFC + 3SS, High-fat and high-cholesterol diet + 3% of *Stachys sieboldii* Miq. root powder; HFC + 5SS, high-fat and high-cholesterol diet + 5% of *Stachys sieboldii* Miq. root powder. Values are means ± standard deviation, *n* = 8. Data were analyzed using one-way ANOVA followed by Tukey’s post hoc test. Means labeled without a common letter differ significantly, *p* < 0.05.

The SS supplementation significantly decreased lipid levels in the blood, liver and adipose tissues through an elevated fecal excretion in the HFC diet-fed male SD rats. Therefore, it is plausible to ask whether SS supplementation could alter the hepatic lipid and cholesterol metabolism at the transcriptional level. The HFC diet considerably escalated the hepatic de novo lipogenic mRNA expression, such as those of acetyl-CoA carboxylase (ACC; Figure 9A) and fatty acid synthase (Fas; Figure 9B). However, as expected, the SS supplementation prevented adipogenic gene expression in the liver without any dose-dependent effect (Figure 9A,B). The elevation of glucose-6-phosphate dehydrogenase (G6pdh) in adiposity increases the level of reactive oxygen species and inflammatory responses. The HFC diet increased the hepatic G6pdh mRNA expression. However, the SS supplementation attenuated the hepatic G6pdh mRNA expression in a dose-dependent manner (Figure 9C). The transcriptional level of cholesterol 7 alpha-hydroxylase (Cyp7a1), the primary and rate-limiting enzyme in bile synthesis, was reduced by the HFC diet compared to that of the RD group. The HFC + 3SS group maintained a similar transcriptional level of Cyp7a1 as the RD group (Figure 9D). Intriguingly, the Cyp1a mRNA expression was significantly increased in the HFC + 5SS group compared to the other groups (Figure 9D). The enzyme 3-hydroxy-3-methyl-glutaryl-coenzyme A reductase (Hmgcr) is a rate-determining and NADH-dependent enzyme for de novo cholesterol synthesis. The HFC and HFC + 3SS groups exhibited higher hepatic transcriptional Hmgcr levels than the RD group. The HFC + 5SS diet further increased the Hmgcr mRNA expression in the liver compared to the HFC or HFC + 3SS diets (Figure 9E). The induction of the hepatic Hmgcr expression may be a compensatory response against the HFC + 5SS diet-induced increased cholesterol excretion. Low-density lipoprotein receptor (Ldlr) is a protein that enables the endocytosis-mediated hepatic cholesterol clearance. We found that the Ldlr mRNA expression was lower in the HFC group than in the RD group (Figure 9F). The HFC + 3SS group maintained a similar hepatic Ldlr mRNA expression level as the HFC group (Figure 9F). However, the transcriptional Ldlr expression in the liver was elevated by the HFC + 5SS group compared to the other groups (Figure 9F).

## 4. Discussion

Obesity (particularly central adiposity) is one of the major health complications of metabolic syndrome (MetS), as well as insulin resistance (IR), hyperglycemia and hypertension [28]. Increased adiposity in obesity is strongly associated with inflammation, oxidative stress and dyslipidemia, which are the factors directly related to the risk of cardiovascular disease and type 2 diabetes mellitus (T2DM) [29,30]. Thus, it is important to develop possible therapeutic strategies in order to prevent the growing obesity epidemic and discover effective therapeutics for treating obesity-related metabolic abnormalities [31]. Although lifestyle modification (such as a well-balanced diet and exercise) is the most important approach to improve the health conditions of patients with obesity and dyslipidemia, its poor adherence rates hinder treatment effectiveness and meaningful outcomes [32]. In addition to the role of lifestyle interventions, pharmacological therapies present a possible option for patients with obesity and dyslipidemia. However, several anti-obesity drugs have been withdrawn from the market due to serious adverse effects. Sibutramine was withdrawn from the market after reports of side effects associated with an increased risk of serious cardiovascular events [33]. Rimonabant was reportedly associated with psychiatric side effects such as mood disturbances and suicidality [34]. Although there are currently 5 FDA-approved prescription medicines for long-term weight management, orlistat, phentermine/topiramate, lorcaserin, naltrexone/bupropion and liraglutide, their potential adverse effects should be taken into consideration [34]. The pharmacological options for dyslipidemia include statins, fibrates, ezetimibe and niacin [35]. While Statins are the first-choice drugs, statin-induced myalgia is the most frequently observed side effect [36]. The second-choice drugs for dyslipidemia include fibrates, ezetimibe and niacin [35]. The reported side effects of fibrates include gastrointestinal disturbances (dyspepsia, nausea, constipation, diarrhea and vomiting) and skin rashes [37]. The adverse effects of using ezetimibe include headache, runny nose and sore throat [37]. The niacin-induced adverse effects include flashing, pruritus, rash, nausea, dyspepsia, abdominal pain and diarrhea [38]. Given the adverse effects and toxicity associated with synthetic drugs for obesity-related dyslipidemia treatment, it is important to develop safer alternatives that could potentially be natural products.

*Stachys sieboldii Miq*. is a medicinal herb belonging to the Lamiaceae family, widely distributed in Asia, North America and Europe [39]. Having a good source of oligosaccharides, proteins and water-soluble vitamins (vitamin B complex), *S. sieboldii* has been extensively used for decades as a folk medicine in China and as a food product in Korea and Japan [17,20]; (S) *sieboldii Miq.* root (SS) reportedly contains several bioactive compounds, including flavonoids, terpenes, phenolic compounds and saponins, known for their antioxidant, anti-inflammatory, antimicrobial and anti-toxic properties [17,40]. Thus, SS has been widely used to treat ischemic stroke, dementia, urinary tract infection, colds, heart disease, tuberculosis and various gastrointestinal problems [18,40]. Although SS is rich in flavonoids and polyphenols, its therapeutic potential for obesity-related dyslipidemia and its related risks for CVD have not yet been elucidated. Obesity is a state of chronic low-grade inflammation. Such an obesity-induced inflammation is highly associated with atherosclerosis [41]. Adipose tissue accumulation in obesity increases the number of macrophages, leading to local inflammation [41]. This leads to the production of proinflammatory cytokines such as tumor necrosis factor-alpha (TNF-α) and interleukin-6 (IL-6) [42]. Macrophage accumulation and local inflammation result in metabolic dysfunction, eventually leading to systemic inflammation and atherosclerosis [39]. High levels of circulating glucose and lipids could increase the production of reactive oxygen species (ROS) [43]. Imbalanced ROS levels lead to increased oxidative stress, which could damage proteins, carbohydrates, lipids and DNA, resulting in several chronic diseases such as cardiovascular disease, diabetes and cancer [42]. Antioxidants protect against such diseases by scavenging excessively produced ROS, inhibiting ROS formation, reducing the oxidation of cellular molecules, alleviating oxidative stress and binding to metal ions [44]. In addition, vitamins C and E, which are highly available in SS, help to prevent the development of oxidative chains by serving as antioxidant radical scavengers [45]. As a good antioxidant source, such as flavonoids and polyphenols, we hypothesized that SS consumption may possess therapeutic potential for obesity-related dyslipidemia and its subsequent risk of CVD.

This study was conducted to evaluate the therapeutic effect of SS in male SD rats, where obesity and dyslipidemia were induced by feeding a high-fat and high-cholesterol (HFC) diet. Obesity is characterized by an excessive amount of body fat and weight caused by imbalanced energy consumption [46]. In order to confirm the anti-obesity effects of SS, we measured BW gain and FER levels. Food intake was measured daily in order to calculate the FER. This study clearly showed that the HFC diet-induced BW gain and FER level increase (Figure 1). Notably, the average BW gain and FER levels significantly decreased in HFC diet-fed rats after SS consumption in a dose-dependent manner. It is widely known that the HFC diet induces hepatomegaly and WAT expansion [47]. Consistent with this, increased liver weight, WAT, MAT, RAT and PAT were observed in all the HFC diet-fed rats (Figure 2). The liver weight and WAT (WAT, MAT, RAT and PAT) showed a trend to be lower in rats fed an SS-supplemented HFC diet. Interestingly, the MAT weight showed the most statistically dynamic change in a dose-dependent manner. The EAT weight was not affected by any of the different diets. However, SS supplementation dose-dependently decreased the EAT size in rats fed the HFC diet (Figure 7). In addition, the increased mRNA expression of the hepatic adipogenic enzymes ACC and FAS was significantly downregulated by the SS supplementation (Figure 9A,B). The consumption of the HFC diet reportedly leads to fatty acid uptake by the liver, resulting in increased liver inflammation and injury [48]. As the serum levels of ALT, AST, ALP and LDH are major hepatic enzymes, we measured their level in order to evaluate how SS could affect the HFC diet-induced hepatocellular abnormality (Figure 3A–D). AST and ALT are biomarkers of liver injury, while ALP and LDH are markers of tumor cell differentiation and necrosis, respectively [49]. As expected, significant increases in serum ALT, AST, ALP and LDH levels were detected in the HFC group. The levels of ALT and AST in the rats fed the SS-supplemented HFC diets were significantly reduced in a dose-dependent manner. Significant decreases in ALP and LDH activities were only observed in rats fed the HFC + 5SS diet. As impaired fasting glucose (Glu) levels could be commonly detected in obesity and dyslipidemia, we also observed elevated levels in rats fed the HFC diet (Figure 3E). The fasting Glu level was significantly decreased only in the HFC + 5SS group. Notably, Glu levels in the HFC + 5SS group were similar to those in the RD group. These results suggest that the SS consumption may improve overt obesity characteristics.

Imbalanced lipid profiles in dyslipidemia are highly associated with residual cardiovascular risk [50]. As atherogenic dyslipidemia (AD) is characterized by elevated TG, TC, non-HDL-C and low HDL-C levels, we measured these lipid parameters, as well as AI and CRF levels in the serum in order to elucidate how SS affects lipid metabolism and CVD risks (Figure 4). Our observation shows that the HFC-induced increased TG, TC and non-HDL-C levels were further attenuated following SS consumption. The HDL-C levels were significantly decreased in rats fed the HFC diets regardless of SS supplementation. Our results demonstrated that SS supplementation may have a protective effect against CVD risk, as increased AI and CRF levels were both significantly attenuated in a dose-dependent manner by supplementing SS in the HFC diet. As we observed a decrease in the serum lipid panel following SS supplementation, we postulated that SS may contribute to reducing fat deposits in the liver and WAT. The hepatic lipid, TG and TC levels increased following the HFC diet (Figure 5). However, the SS supplementation in the HFC diet significantly reduced the hepatic TG and TC levels. Interestingly, the hepatic TC level in rats fed HFC + SS decreased in a dose-dependent manner. The beneficial effect of SS was confirmed through fat cell- and neutral fat histology using Oil Red O staining. Consistent with the results of the hepatic TG and TC level measurements, the SS-supplemented HFC groups exhibited smaller lipid droplets. Among the WATs, EAT and MAT were particularly selected as the EAT weight was not affected by the SS supplementation, while the MAT weight significantly decreased following SS supplementation. In the EAT, the total lipid levels showed a trend to decrease, similar to those in the RD group following SS supplementation in the HFC group (Figure 6). This trend may be associated with the EAT weight that was not altered by SS consumption. Consistent with the EAT size, the TG and TC levels in the EAT were dose-dependently reduced following the SS supplementation. Consistent with the results from the weight of the MAT, the increased levels of total lipids, TG and TC in the HFC group were attenuated with increasing SS levels. Therefore, SS may have protective effects against excessive fat accumulation in the liver and the WATs. As SS supplementation significantly decreased lipid levels in the serum, liver and adipose tissues, we have expected to observe increased fecal fat excretion. While HFC did not affect the fecal lipid levels, the SS supplementation dramatically increased the fecal lipid excretion in a dose-dependent manner (Figure 8). The levels of fecal TG were not altered by the different diets. As the HFC diet contains higher levels of cholesterol, greater fecal cholesterol excretion was expected in rats following the HFC diet. Interestingly, we detected a significant increase in fecal cholesterol excretion in rats fed HFC + 5SS diets. Thus, SS may contribute to lipid level reduction in the blood, liver and adipose tissues through increased fecal fat and cholesterol excretion. In addition, we hypothesized that SS supplementation could change lipid metabolism at the transcriptional level. Increased mRNA levels of G6pdh, a rate-limiting enzyme of the pentose phosphate pathway (PPP), are reportedly associated with adipose tissue inflammation [51]. As commonly seen in obesity, the hepatic G6pdh mRNA expression was significantly increased following the HFC diet (Figure 9C–F). However, the SS supplementation dose-dependently attenuated the G6pdh mRNA expression. The cholesterol homeostasis is regulated by Cyp7a1 and Hmgcr. Cyp7a1 is the rate-limiting enzyme that catalyzes the initial step in cholesterol catabolism and bile acid synthesis [52]. The HFC diet significantly decreased the Cyp7a1 mRNA level, while the SS-supplemented HFC diet significantly increased it. As a result, hepatic cholesterol levels decreased, and the bile acid pool increased in SS-supplemented HFC diet-fed rats compared with HFD-fed rats. Hmgcr is the rate-controlling enzyme of the mevalonate pathway for de novo cholesterol synthesis [53]. This study showed that the HFC and HFC + 3SS diets upregulated the Hmgcr mRNA levels. Interestingly, the HFC + 5SS diet further increased the Hmgcr mRNA expression. The increased Hmgcr expression in the SS-supplemented HFC diet may be associated with a compensatory response to increased fecal cholesterol excretion. Ldlr is a mosaic protein with an important role in the endocytosis-mediated plasma lipid clearance [54]. The HFC diet increased hepatic Ldlr mRNA expression. The HFC + 3SS diet resulted in similar levels of hepatic Ldlr to those of the HFC diet. However, the HFC + 5SS diet caused the greatest increase in hepatic Ldlr expression.

Taken together, the results of this study demonstrated that the *Stachys sieboldii Miq*. root (SS) powder (especially at 5%) exhibits anti-obesity and anti-dyslipidemic effects in rats following a high-fat and high-cholesterol (HFC) diet for 6 weeks. The anti-obesity effects were proven by the reduced BW, liver weight, various adipose tissue weights and sizes, hepatic adipogenic gene expression, hepatocellular injury and fasting glucose levels. The anti-dyslipidemic effects were indicated by the improved lipid profile and reduced lipid accumulation in the serum, liver and adipose tissues, as well as by the reduced CVD risk parameters, increased fecal lipid excretion and enhanced lipid metabolism at the transcriptional level. Our study demonstrated that SS may possess therapeutic potential for treating obesity-related dyslipidemia and its subsequent risk of CVD. Future studies may focus on the health benefits of SS-derived dietary fibers. Despite high fiber content, the effects of SS on obesity and dyslipidemia have not yet been evaluated [55,56]. As SS was extensively used as folk medicine and in food products for decades with no reported adverse effects, we postulate that it may be considered as a relatively safe material. However, further studies need to confirm its clinical safety.

## 5. Conclusions

In conclusion, the results obtained from this study support our hypothesis that *Stachys sieboldii Miq*. root (SS) powder may possess anti-adipogenic and lipid-reducing effects by enhancing lipid metabolism. Our data suggest that SS may be used as a therapeutic alternative to synthetic drugs for the treatment of obesity-related dyslipidemia.

## Figures and Tables

**Table 1:** Composition of experimental diet.

	Groups	RD ^(1)^	HFC ^(2)^	HFC + 3SS	HFC + 5SS
Diet Composition (g)					
Casein		200	200	200	200
L-cystine		3	3	3	3
Corn starch		397.486	287.486	257.486	237.486
Dextrose		132	132	132	132
Sucrose		10	10	10	10
Cellulose		50	50	50	50
Lard			100	100	100
Soybean oil		70	70	70	70
Cholesterol			10	10	10
Mineral mix ^(3)^		35	35	35	35
Vitamin mix ^(4)^		10	10	10	10
Choline chloride		2.5	2.5	2.5	2.5
*tert*-Butylhydroquinone		0.014	0.056	0.056	0.056
*Stachys sieboldii Miq.* root powder		0.0	0.0	30	50
Total (g)		1000.0	1000.0	1000.0	1000.0
Total energy (kcal)		3999.9	4549.9	4429.9	4349.9
Fat (kcal%)		15.8	35.6	36.6	37.2

^(1)^ RD: regular diet; ^(2)^ HFC: high-fat and high-cholesterol diet; ^(3),(4)^ AIN-93-GX mineral mixture and AIN-93-VX vitamin mixture.

**Table 2:** RT-PCR primer sequences (5′ to 3′).

Transcript	Forward Primer	Reverse Primer
Acc	CAACGCCTTCACACCACCTT	AGCCCATTACTTCATCAAAGATCCT
Fas	GGAACTGAACGGCATTACTCG	CATGCCGTTATCAACTTGTCC
G6pdh	GTTTGGCAGCGGCAACTAA	GGCATCACCCTGGTACAACTC
Cyp7a1	GCCGTCCAAGAAATCAAGCAGT	TGTGGGCAGCGAGAACAAAGT
Hmgcr	GTGATTACCCTGAGCTTAGC	TGGGATGTGCTTAGCATTGA
Ldlr	ATTTTGGAGGATGAGAAGCAG	CAGGGCGGGGAGGTGTGAGAA
β-actin	GTGGGGCGCCCCAGGCACCAGGGC	CTCCTTAATGTCACGCACGATTTC
